# Feasibility and Safety of Drug-Coated Balloon-Only Angioplasty for De Novo Ostial Lesions of the Left Anterior Descending Artery: Two-Center Retrospective Study

**DOI:** 10.3389/fcvm.2022.874394

**Published:** 2022-04-25

**Authors:** Chuang Li, Xuebo Ding, Lefeng Wang, Kuibao Li, Xinchun Yang, Liping Liu, Li Xu

**Affiliations:** ^1^Heart Center and Beijing Key Laboratory of Hypertension, Beijing Chaoyang Hospital, Capital Medical University, Beijing, China; ^2^Heart Center, Sinopharm Tongmei General Hospital, Shanxi, China

**Keywords:** DCB-only angioplasty, *de novo* ostial coronary lesions, coronary artery disease, adverse clinical events, intervention

## Abstract

**Introduction:**

There is limited evidence regarding the effectiveness of drug-coated balloon (DCB) angioplasty in the percutaneous treatment of complex *de novo* ostial coronary lesions. This study primarily aimed to explore the feasibility and safety of this innovative approach for ostial lesions in the left anterior descending artery (LAD).

**Methods:**

Patients treated with paclitaxel DCB or second-generation drug-eluting stents (DES) were retrospectively enrolled from two large centers. The primary endpoints were major adverse cardiovascular events (MACE), including cardiovascular death, target lesion revascularization (TLR), target vessel revascularization, and recurrent myocardial infarction related to target artery occlusion. Cox regression analysis was used to identify risk factors for MACE, and propensity score matching was performed to minimize selection bias.

**Results:**

A total of 388 patients were included; among them, 52 were treated with paclitaxel DCB, and 336 with DES for ostial LAD lesions. Using propensity score matching, 49 patients were treated with DCB-only and 49 with the DES strategy. The average follow-up time was 12 months; subsequently, a non-significant decrease in MACE rate was observed in the DCB-only angioplasty treatment group (MACE: 6 vs. 6%, *p* = 1.0; TLR: 2 vs. 4%, *p* = 0.56). Cox regression analysis indicated that DCB-only angioplasty was not an independent risk factor for adverse events after adjusting for confounding risk factors (hazard ratio: 1.713, *p* = 0.43).

**Conclusion:**

The use of the DCB-only approach is an innovative and optional strategy in the treatment of isolated ostial LAD disease. A further randomized trial is of necessity to confirm the feasibility and safety of drug-coated balloon-only angioplasty for LAD *de novo* ostial lesions.

## Introduction

An isolated ostial lesion of the left anterior descending artery (LAD) is an unusual symptom of coronary artery disease. In intravascular ultrasound studies, the specific location and potential involvement of the distal left main (LM) artery have been observed; (1, 2) therefore, the LM artery cross-over technique is generally recommended for the treatment of LAD ostial lesions in the era of stenting. Previous studies found that cross-over LM stenting for ostial LAD has highly favorable clinical outcomes; however, almost 50% required restenosis in the LM segment adjacent to the stent (3, 4). Meanwhile, cross-over stenting has become a major contributor to the flow limitation and risk of acute occlusion or late stenosis of the left circumflex (LCX) artery ostium because stenting inevitably covers the structure (5–7). Current clinical studies have yet to identify the optimal strategy for the treatment of isolated LAD ostial lesions.

A drug-coated balloon (DCB) is an emerging angioplasty technique. It combines mechanical expansion of the vessel and reduction of neointimal hyperplasia without implanting protruded struts. This eliminates the risk of late or extremely late stent thrombosis and reduces the duration of dual antiplatelet treatment (8). A recent SPARTAN DCB study found that paclitaxel DCB for *de novo* coronary artery disease had comparable long-term mortality rates with second-generation drug-eluting stents (DES) (9). However, previous studies generally recommended DCB for the treatment of in-stent restenosis (ISR) and small-vessel disease (diameter <2.5 mm) (10, 11). Rosenberg et al. reported that DCB-only percutaneous coronary intervention (PCI) was equally effective against large coronary arteries with *de novo* lesions and small vessel disease (12). Currently, the available data do not support the broad use of DCB for large *de novo* lesions, especially those that involve the ostium (13). Therefore, this study aimed to explore the safety and efficacy of the use of DCB in lesions at the LAD ostium.

## Materials and Methods

### Study Population

This retrospective study was conducted in two different cardiac centers, Beijing Chaoyang Hospital and Sinopharm Tongmei General Hospital, between January 2017 and December 2019. This study included patients with *de novo* lesions at the LAD ostium who refused to undergo second-generation DES and received SeQuent Please DCB angioplasty due to symptomatic coronary artery disease. Meanwhile, patients with *de novo* LAD ostium lesions undergoing second-generation DES were recruited during the same period. Given that coronary artery bypass graft to LAD could influence the outcome, patients with history of coronary artery bypass grafting were excluded. The other exclusion criteria were end-stage renal disease, ISR, and/or a left ventricular ejection fraction <35%. The study was performed in accordance with the Declaration of Helsinki and its later amendments and was approved by the local ethical review board of the two hospitals.

### Lesion Preparation and Interventional Procedure

Angiography and angioplasty were performed by two independent senior physicians (Dr. Liu and Pro. Xu). The distance between the lesions and the LAD ostium was confined to 3 mm. Ostial LAD lesions with significant left main coronary artery (LMCA) distal bifurcation stenosis were regarded as LMCA lesions and excluded from the current analysis. Based on the Medina classification system, the bifurcation lesions in this study only involved (0, 1, 0) and (0, 1, 1) lesions. Fluoroscopy time (FT) and dose-area product (DAP) were recorded. All angiograms were analyzed using a quantitative coronary angiographic system (QCA, CASS system). Percent diameter stenosis, reference vessel diameter, and minimal luminal diameter were measured pre- and post-intervention.

**Figure 1:**
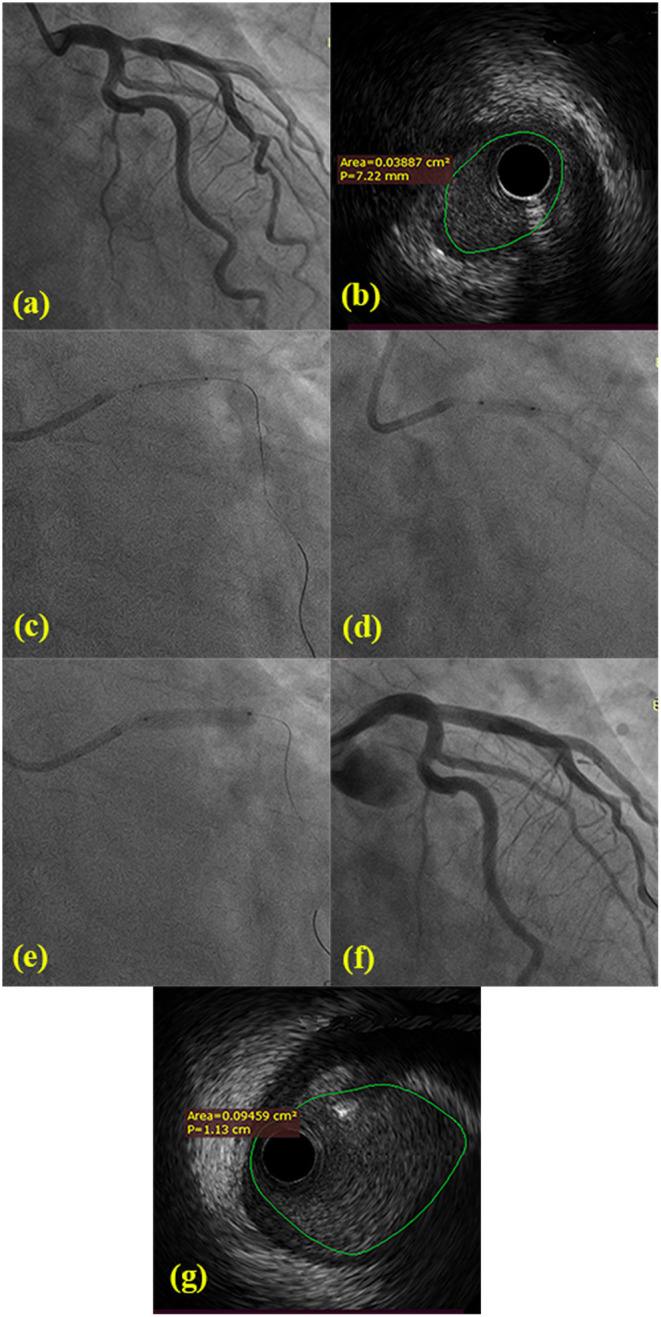
**(a)** Angiology showing 80% ostial stenosis of LAD and normal LMB and LCX from two different angles. **(b)** Pre-procedural IVUS confirmed the minimal lumen area of LAD ostial lesions. **(c)** The 2.0 mm × 15 mm balloon was pre-dilated at LAD ostial lesions. **(d)** The 3.0 mm × 10 mm cutting balloon was repeatedly predilated at LAD ostial lesions. **(e)** The 3.5 mm × 20 mm DCB was dilated with 7 atm × 60 s. **(f)** The post-procedural angiology showing significant stenosis decreasing. **(g)** Post-procedural IVUS confirmed the minimal lumen area and the extent of dissection following DCB-only.

DCB angioplasty (SeQuent Please, B.Braun Interventional Group, Ltd, Melsulgen, Germany) with a bailout stenting strategy was conducted according to international guidelines and the current consensus (14). Target lesions were pretreated using standard balloon angioplasty, a non-compliant balloon, and/or cutting balloons. After achievement of minimal residual stenosis, the DCB (recommended diameter was 0.8–1.0:1 of nominal target vessel size) could be inflated for 30–90 s. In cases of residual stenosis >50% or type C coronary dissections, a stent was implanted only as a bailout for the treatment of suboptimal results after DCB. Intravascular ultrasonography (IVUS) was not routinely performed. IVUS imaging was conducted by a commercial scanner (Boston Scientific Corporation, Marlborough, MA) and performed after intracoronary administration of 0.2 mg nitroglycerin to identify the gradient of calcification, measure external elastic membrane area and the minimum lumen area of major and side branch pre- and post-intervention, and provide a precise diameter reference (as shown in Figure 1).

Stent implantation was performed using a standard procedure according to the type of lesion. Stenting approaches were performed at the operator's discretion: cross-over stenting from the LM across the LAD ostium into the diseased branch or stenting right at the ostium of the diseased branch. Regarding the cross-over strategy, a bifurcation technique with a provisional side branch was implemented. The final kissing balloon was routinely used in patients treated with cross-over stenting. No strict protocol on how or which interventionist should perform the procedure was present, and there was also no restriction on the choice or kind, or the numbers of stents deployed. The following thin-strut stents were used: Resolute (Medtronic CardioVascular, Santa Rosa, CA), Xience (Abbott Vascular Devices, Santa Clara, CA), Promus (Boston Scientific, Natick, MA), Excel (JW Medical Systems, Shandong province, CN) (as shown in Supplementary Table).

### Study Outcomes and Follow-Up

The primary outcomes were defined as major adverse cardiovascular events (MACE), including cardiovascular death, target lesion revascularization (TLR), target vessel revascularization (TVR), and recurrent myocardial infarction related to target vessel occlusion. Adjudication of adverse outcomes was performed by two physicians (Dr. Ding X and Li K) not involved with the interventional procedures. TLR and TVR were defined in accordance with the definitions of end points for clinical trials (15). Angiographic follow-up was only scheduled if non-invasive evaluation or clinical presentation suggested the presence of ischemia. All patients were followed-up through telephone interviews, outpatient visits, or hospital records.

### Statistical Analysis

Statistical analysis was performed using the STATA system (15.0 version). Categorical and continuous variables are presented as frequency (percentage) and mean ± standard deviation, respectively. Comparisons between groups were performed using the independent Student's *t*-test or Mann-Whitney test for continuous variables and the χ^2^ test or Fisher's exact test for categorical variables. Kaplan-Meier survival curves were constructed to compare the incidence of MACE among the groups. Comparisons were performed using log-rank tests. Cox regression analysis was used to identify the role of intervention strategy as an independent risk factor for MACE. A 1:1 nearest-neighbor propensity score matching with a default caliper distance of 0.25 was also performed to minimize selection bias. Variables for matching included age, sex, history of myocardial infarction, hypertension, diabetes mellitus, hyperlipidemia, PCI, ejection fraction, and target vessel diameter. All tests performed were two-tailed, and statistical significance was set at *p* < 0.05.

## Results

### Baseline Clinical Characteristics

**Figure 2:**
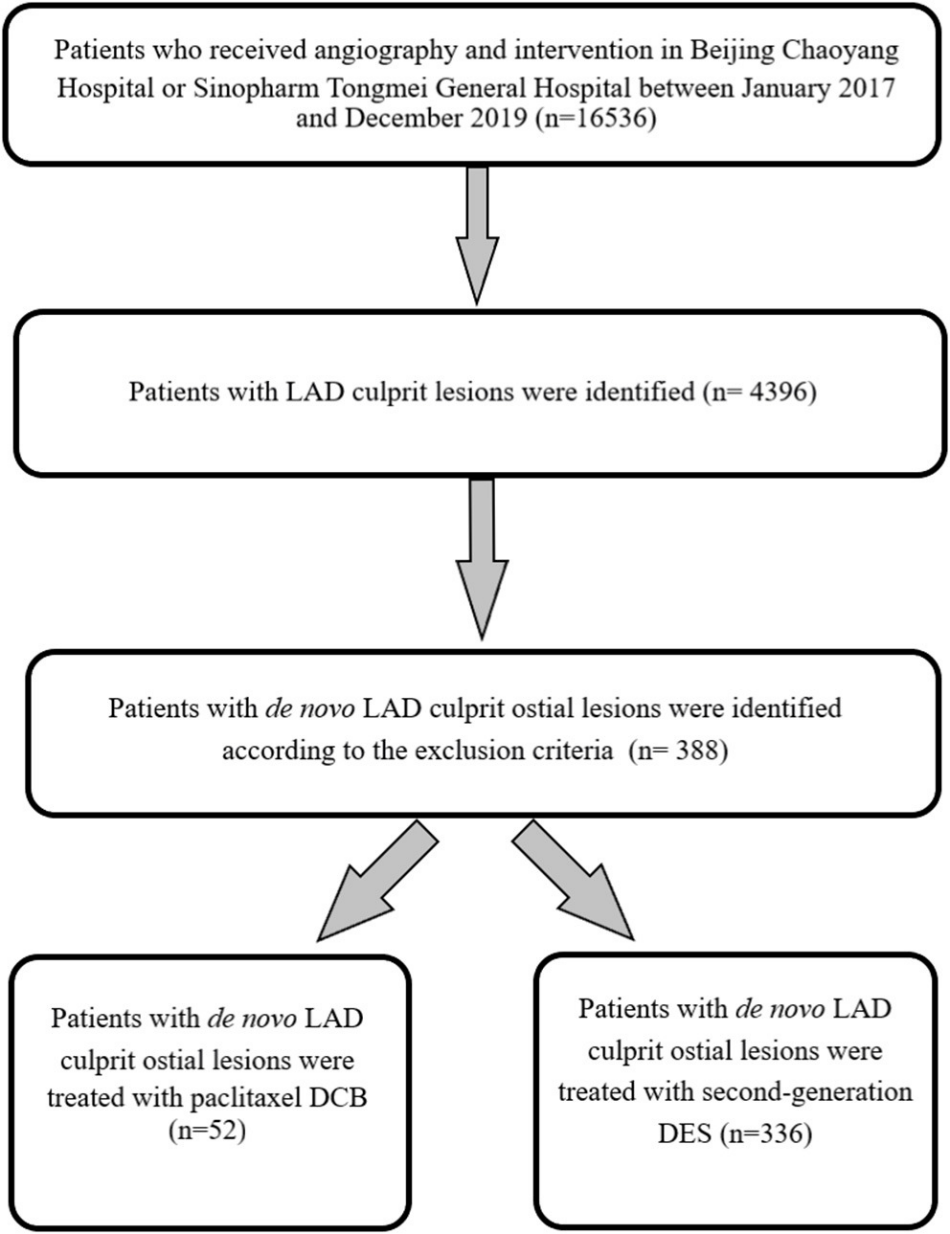
The flowchart of this study.

Between January 2017 and December 2019, 16,536 patients underwent angiography and intervention. Based on the exclusion criteria, a total of 388 consecutive patients with LAD culprit ostial lesions, including 52 patients treated with paclitaxel DCB and 336 patients with second-generation DES, were finally recruited (shown in Figure 2). Compared to patients treated with DCB, those with DES had more frequent histories of myocardial infarction, and higher values of SYNTAX and SYNTAX II scores. After propensity score matching, 49 patients were treated using DCB-only and another 49 with DES. The two treatment groups were well-balanced in terms of baseline demographic and clinical characteristics (Table 1). However, patients in the DES group had significantly higher SYNTAX scores and shorter follow-up duration (*p* < 0.05).

**Table 1:** Baseline characteristics.

	**Before propensity matching**			**After propensity matching**		
	**DES**	**DCB**	***p*-value**	**DES**	**DCB**	***p*-value**
*N*	336	52		49	49	
Age, year	61 (53, 71)	62 (54, 69)	0.66	61(55, 75)	62 (54, 68)	0.30
Sex, *n* (%)	268 (79.8%)	37 (71.2%)	0.20	35 (71%)	37 (76%)	0.82
Previous MI, *n* (%)	75 (22.3%)	1 (1.9%)	<0.001	3 (6%)	1 (2%)	0.31
Previous PCI, *n* (%)	87 (25.9%)	8 (15.4%)	0.10	6 (12%)	8 (16%)	0.56
DM, *n* (%)	129 (38.4%)	13 (25.0%)	0.062	12 (24%)	12 (24%)	1.00
Hypertension, *n* (%)	183 (54.5%)	24 (46.2%)	0.26	21 (43%)	23 (47%)	0.68
Hyperlipemia, *n* (%)	180 (53.7%)	15 (28.8%)	<0.001	17 (35%)	15 (31%)	0.67
Smoker, *n* (%)	240 (71.4%)	22 (42.3%)	<0.001	30 (61%)	22 (45%)	0.16
Creatinine, mmol/L	83.47 (72.58)	70.72 (14.3)	0.21	66.2 (15.34)	71.1 (14.72)	0.11
EF, %	54.76 (12.38)	60.67 (9.83)	0.001	58.1 (12.2)	60.1 (9.84)	0.36
SYNTAX score	24.66 (8.67)	17.36 (6.92)	<0.001	20.9 (8.17)	17.6 (7.00)	0.04
SYNTAX score II	30.56 (11.07)	25.02 (6.71)	<0.001	26.1 (7.34)	24.8 (6.84)	0.39

*Continuous variables were presented as mean (SD) or median (IQR) and categoric variables were shown as n (%). MI, myocardial infarction; PCI, percutaneous coronary intervention; DM, diabetes mellitus; EF, ejection fraction*.

### Angiographic Characteristics and Quantitative Coronary Analysis

The characteristics of angiography, procedure, and details of the QCA are presented in Table 2. No significant difference was observed regarding the lesions found in the LMCA, LAD, and LCX arteries between the two groups. According to the angiographic characteristics, the DCB group tended to have a lower frequency of multivessel disease and calcified lesions (61 vs. 41%, *p* = 0.07, and 10 vs. 2%, *p* = 0.09, respectively). Among all the procedures, 64% of DES patients and all DCB patients underwent the cross-over technique (*p* < 0.001). On the other hand, the DCB group had significantly lower FT and DAP than those of the DES group (*p* < 0.001). In the DCB treatment group, 12 patients had unstable hemodynamics; among them, one had a C-type dissection while 11 experienced hypotension which disappeared through coughing during the DCB dilatation. The QCA data revealed that the DCB group had significantly smaller post-procedural minimal lumen diameter (2.78 ± 0.38 mm vs. 3.10 ± 0.39 mm, *p* < 0.001) and higher diameter stenosis (33.05 ± 11.88% vs. 19.78 ± 10.75%, *p* < 0.001); on the other hand, the DES group had significantly more aggressive targeted artery stenosis (91.21 ± 6.47 vs. 87.94 ± 7.55%, *p* = 0.03).

**Table 2:** The angiographic and procedural characteristic of quantitative coronary angiography analysis.

**Factor**	**DES group**	**DCB group**	***p*-value**
*N*	49	49	
**Angiographic feature**			
Multivessel disease, *n* (%)	30 (61%)	20 (41%)	0.07
Bifurcation angle >90°, *n* (%)	40 (82%)	45 (92%)	0.23
Calcified lesion, *n* (%)	5 (10%)	1 (2%)	0.09
**Medina classification**, ***n*** **(%)**			0.81
(0, 1, 1)	12 (24%)	10 (20%)	
(0, 1, 0)	37 (76%)	38(78%)	
**Procedural characteristic**			
Cross-over, *n* (%)	30 (64%)	49 (100%)	<0.001
IVUS, *n* (%)	-	5 (10.2%)	-
Type-C dissection, *n* (%)	-	1 (2.0%)	-
Blood pressure descent, *n* (%)	-	11 (22.4%)	-
DAP, Gy/cm2	187 (140, 236)	94 (84.3, 105)	<0.001
Intervention time, minutes	90 (75, 108)	65 (56, 78)	<0.001
**Previous intervention**			
LMCA RVD, mm	4.30 (0.75)	4.16 (0.72)	0.35
LMCA MLD, mm	3.88 (0.65)	3.79 (0.79)	0.54
LMCA, %	16.75 (13.72)	16.33 (16.48)	0.89
LAD RVD, mm	3.52 (3.22, 3.63)	3.44 (3.04, 3.63)	0.33
LAD MLD, mm	0.96 (0.44)	1.11 (0.51)	0.13
LAD DS, %	91.21 (6.47)	87.94 (7.55)	0.03
TL length, mm	22.03 (10.26)	19.15 (5.69)	0.10
LCX RVD, mm	3.07 (0.60)	3.14 (0.71)	0.60
LCX MLD, mm	2.73 (0.67)	2.80 (0.72)	0.62
LCX DS, %	20.74 (17.62)	20.53 (15.77)	0.95
**Post intervention**			
LM RVD, mm	4.35 (0.73)	4.14 (0.66)	0.17
LM MLD, mm	3.99 (0.70)	3.88 (0.65)	0.48
LM DS, %	14.73 (14.01)	11.06 (10.58)	0.18
LAD RVD, mm	3.50 (3.21, 3.65)	3.35 (3.03, 3.57)	0.12
LAD MLD, mm	3.10 (0.39)	2.78 (0.38)	<0.001
LAD DS, %	19.78 (10.75)	33.05 (11.88)	<0.001
LCX RVD, mm	3.05 (0.64)	3.08 (0.66)	0.81
LCX MLD, mm	2.68 (0.64)	2.78 (0.73)	0.53
LCX DS, %	25.01 (17.79)	19.01 (17.25)	0.12
Follow-up	(*n* = 13)	(*n* = 16)	
LM RVD, mm	4.20 (0.83)	4.08 (0.73)	0.66
LM MLD, mm	4.08 (0.85)	3.89 (0.77)	0.53
LM DS, %	2.3 (0.9, 11.0)	9.5 (0.3, 13.2)	0.41
LAD RVD, mm	3.43 (0.49)	3.38 (0.49)	0.79
LAD MLD, mm	3.0 (0.50)	2.6 (0.93)	0.14
LAD DS, %	22.3 (11.8, 30.6)	25.8 (22.4, 35.4)	0.18
LCX RVD, mm	2.95 (0.71)	3.00 (0.74)	0.87
LCX MLD, mm	2.58 (0.74)	2.64 (0.77)	0.83
LCX DS, %	23.4 (17.9, 31.3)	20.7 (12.7, 31.2)	0.59

*Continuous variables were presented as mean (SD) or median (IQR) and categoric variables were shown as n (%). DCB, Drug-coated balloon; DES, Drug-eluting stents; DAP, dose-area product; LM, left main; LMCA, left main coronary artery; LAD, left anterior descending artery; LCX, left circumflex artery; RVD, reference vessel diameter; MLD, minimal lumen diameter; DS, diameter stenosis; IVUS, intravascular imaging*.

### Clinical Outcomes

A significant difference was observed regarding the follow-up duration between the DES (16 months) and DCB (12 months) groups (Table 3) (*p* < 0.001). However, no significant difference was observed regarding the all-cause mortality and MACE incidence between the two groups (MACE: 11.3 vs. 3.8%, p=0.10; all-cause mortality: 3.3 vs. 0.0%, *p* = 0.19). In the DCB group, two recurrent myocardial infarctions due to acute vessel closure and two TLRs were observed.

**Table 3:** The cumulative incidence of MACE.

	**Before propensity matching**			**After propensity matching**		
	**DES**	**DCB**	***p*-value**	**DES**	**DCB**	***p*-value**
N	336	52		49	49	
Time, month	16 (8.7)	12 (2.4)	<0.001	13 (3.0)	12 (2.6)	0.54
MACE Total, *n* (%)	38 (11.3%)	3 (3.8%)	0.10	3 (6%)	3 (6%)	1.0
Cardiovascular mortality, *n* (%)	11 (3.3%)	0 (0.0%)	0.19	1 (2%)	0 (0%)	0.31
MI related to target vessel occlusion, *n* (%)	9 (2.7%)	2 (3.8%)	0.75	0 (0%)	2 (4%)	0.15
TVR, *n* (%)	25 (7.4%)	0 (0.0%)	0.04	2 (4%)	0 (0%)	0.15
TLR, *n* (%)	5 (1.5%)	2 (3.8%)	0.23	1 (2%)	2 (4%)	0.56

*MACE, major adverse cardiovascular events; MI, myocardial infarction; TVR, target vessel revascularization; TLR, target lesion revascularization*.

In the propensity score analysis (49 patients in each group), the overall event rates in both groups were insufficient to find significant differences regrading adverse outcomes between the two groups (MACE: 6 vs. 6%, *p* = 1.0). The MACE rate was relatively low in the DCB-only angioplasty treatment arm and was mainly triggered by post-procedure TLR.

### Cox Hazard Regression and Kaplan-Meier Survival Curves

**Figure 3:**
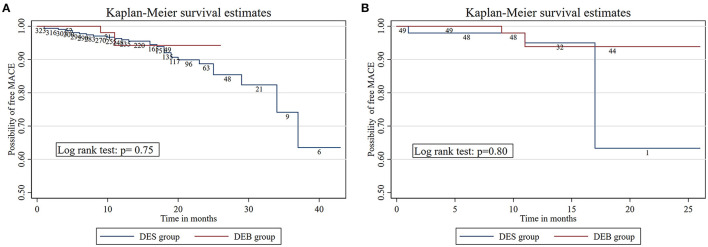
**(A)** The Kaplan-Meier survival estimates between DES group and DCB group before neighboring propensity score matching. **(B)** The Kaplan-Meier survival estimates between DES group and DCB group after neighboring propensity score matching.

Paclitaxel DCB for *de novo* coronary artery lesions of the ostium did not increase the incidence of adverse events. Kaplan-Meier survival curves indicated that the cumulative incidence of MACE was similar between the two groups after propensity score analysis (log-rank test, *p* = 0.80; shown in Figure 3). Meanwhile, Cox regression analysis demonstrated that the strategy of angioplasty was not an independent risk factor for adverse events following the treatment of ostial LAD lesions (HR = 1.713, 95% CI = 0.456–6.441, *p* = 0.43) (Table 4).

**Table 4:** Cox proportional hazard regression analyses for major adverse cardiovascular events.

	**Univariate analysis**		**Multivariable analysis**	
	**HR (95%CI)**	***P***	**HR (95%CI)**	***P***
DCB strategy	1.222 (0.353, 4.227)	0.75	1.713 (0.456, 6.441)	0.43
Age	1.014 (0.987, 1.043)	0.30	1.014 (0.985, 1.044)	0.34
Male	1.086 (0.424, 2.353)	0.99	0.926 (0.374, 2.289)	0.87
History of PCI	2.228 (1.089, 4.557)	0.02	2.662 (0.767, 9.243)	0.12
History of MI	1.954 (0.918, 4.162)	0.08	1.037 (0.251, 4.279)	0.96
Diabetes mellitus	1.191 (0.582, 2.437)	0.63	0.893 (0.410, 1.943)	0.78
Hypertension	0.881 (0.435, 1.783)	0.73	0.763 (0.357, 1.629)	0.49
Hyperlipemia	1.153 (0.567, 2.344)	0.69	1.044 (0.458, 2.385)	0.92
Current smoking	1.170 (0.757, 1.807)	0.48	0.916 (0.547, 1.532)	0.74
EF	0.987 (0.958, 1.017)	0.40	0.982 (0.950, 1.017)	0.34
Creatinine	1.006 (0.997, 1.016)	0.19	1.009 (0.998, 1.020)	0.08
SYNTAX	1.012 (0.976, 1.050)	0.52	1.017 (0.978, 1.058)	0.39

*DCB, drug coated balloon; PCI, percutaneous coronary intervention; MI, myocardial infarction; EF, ejection fraction*.

## Discussion/Conclusion

Guidelines and previous studies have supported the effectiveness and safety of DCB-only angioplasty for the treatment of ISR and small and large *de novo* coronary artery disease (9, 12, 16, 17). This retrospective two-center study primarily found similar results between the treatment of DCB-only and DES strategy on LAD ostium lesions observed during the average median of 12 months. Therefore, this study provides evidence regarding the feasibility and safety of DCB in special LAD ostial lesions.

The complex geographic features and high technical skill requirement increase the difficulty of performing percutaneous procedures for ostial lesions. Inaccurate stent localization at the LAD focal ostium inevitably increases the risk of plaque shift into the LM and/or LCX and for distal LM damage related to balloon dilatation, which results in the placement of the LM segment adjacent to the stent, stimulating a progressive restenosis lesion (18). On the other hand, deploying a cross-over stent in the LAD ostium potentially increases the risk of recurrent restenosis or stent thrombosis in the setting of a normal LM coronary artery. Occasionally, there is the possibility of bail-out two stent technologies that are susceptible to a higher risk of in-stent stenosis. Previous studies found that left main bifurcation lesions and involvement are independent risk factors for in-stent restenosis (19, 20). In contrast, no progression of LCX stenosis and minimal lumen diameter were observed in the DCB group, which is consistent with the results of previous studies (21, 22). Further, an absolute decrease in DAP values (93 Gy/cm^2^) was observed for patients receiving the DCB-only approach instead of DES. This significant difference indicates that the DCB-only strategy lowers ionizing radiation exposure time and dose. Considering these results, DCB-only angioplasty protects the LCX ostium and simplifies the complex cross-over stent technique, which involves guidewire or jailed balloon side branch protection, postprocedural rewiring, kissing balloon, and the proximal optimization technique. Furthermore, DCB-only angioplasty decreases ionizing radiation exposure time and dose, which may likely impair the cardiologists' and patients' retina and increase the risk of tumors (23, 24).

Previously, interventional strategies with coronary stenting remained prevalent because it eliminates lesion recoil and reocclusion of the infarct artery related to balloon angioplasty. However, Brodie et al. recently found similar trends for 1-year cardiovascular mortality and reinfarction rates between patients with acute myocardial infarction treated with a bare metal stent or DES (25), which is similar to our findings. The efficacy and safety of DCB with SeQuent Please in the treatment of native-vessel coronary artery disease have been reported (12, 13, 26). The BASKET-SMALL 2 is the largest randomized clinical study that compared the Sequent Please DCB-only approach with everolimus or Taxus DES in the treatment of small *de novo* coronary arteries (<3 mm); the study found that DCB was not inferior to DES in terms of MACE (7.5 *vs*. 7.3%). Additionally, the DCB group had a non-significantly higher risk of cardiac death than the DES group (3.1 vs. 1.3%) during the 12-month follow-up (27). Furthermore, previous studies demonstrated that the Sequent Please DCB-only strategy has comparable safety and effectiveness for *de novo* lesions of large coronary vessels (12, 28, 29). The recent large-scale DEB-Dragon-Registry (17) indicated that similar favorable clinic outcomes were observed between the new generation DCB and thin-DES used in the current study for patients with ISR. Regarding complex bifurcation, Kook et al. (30) recently reported comparable long-term clinical outcomes of DES and Sequent Please DEB in patients with ISR involving the complex LMCA bifurcation. This study also found that DCB angioplasty had rates of adverse clinical outcomes similar to the results of the DEB-Dragon-Registry and Kook et al. The incidence of target vessel revascularization in our study indicated that the DCB-only approach for isolated *de novo* LAD-ostium angioplasty potentially avoids late and extremely late in-stent thrombosis, reduces early restenosis and neo-atheroma formation, and simplifies the procedure.

DCB of *de novo* lesions has several advantages; however, acute vessel closure due to elastic recoil and flow-limiting dissections can restrict DCB. Particularly, the BASKET-SMALL 2 (27) study found that 14% of cases failed due to dissection or residual stenosis following predilatation. This rare but fatal immediate side effect of the DCB-only approach should be taken into consideration. In the present study, only one patient (2%) in the DCB group developed acute occlusion and received a bailout stent in the ostial LAD. These favorable results may be attributable to the decreased calcified lesions selected by experienced operators. In the pathogenesis of ACS, the calcified plaque is the least frequent etiology, but is associated with aggressive lesion preparation, which necessitates predilatation using a high-pressure post, large-sized, or cutting balloon (31, 32). This approach may achieve a sufficient lumen area but increases the risk of coronary dissection. In the study by Sugiyama et al. (33) a superficial calcific sheet, a type of calcified plaque, more commonly affects the LAD and needs debulking or plaque modification such as rotational and orbital atherectomy. Similar to the other studies (12, 34–36), the middle-term TLR rates were as low as 4% despite approximately 30% residual stenosis of the target coronary artery. Completing an uncompact predilatation prior to the DCB-only treatment approach potentially contributes to subsequent stenting implantation and increased TLR rates (12, 37). Hence, it is worth noting that rigorous lesion preparation to achieve an acceptable angiographic result before the use of DCB is crucial to avoid complications.

### Limitations

This study has some limitations. First, its retrospective and non-randomized nature and the relatively small sample size may introduce unavoidable referral bias. However, practitioners from these two centers located in different districts performed more than a thousand cardiac interventions annually, which minimizes selection bias. Second, the accuracy of lumen measures and further comparisons in all details were confined to a relatively low proportion of IVUS conducted; additionally, follow-up angiography was not performed. Nevertheless, this study was able to provide real-word data that underwent propensity score matching, which could increase the credibility of data analysis. Moreover, there was no precise protocol on how an intervention should be performed or which interventionist should perform the procedure. These two different stenting approaches (crossover stenting and just proximal stenting) potentially influenced clinic outcomes and led to confounding bias. Finally, this current analysis excluded moderate or severe patients with calcification and had a lower proportion of complicated bifurcation lesions. This selection bias may have resulted in favorable outcomes and generalizability limitations.

### Conclusions

As the current study presented, the use of the DCB-only approach represents an optional strategy in the treatment of *de novo* lesions at the ostium of the LAD, especially in patients with high bleeding risk or with indication to short DAPT duration. A further randomized trial is of necessity to confirm the feasibility and safety of drug-coated balloon-only angioplasty for LAD *de novo* ostial lesions.

## Data Availability Statement

The raw data supporting the conclusions of this article will be made available by the authors, without undue reservation.

## Ethics Statement

The studies involving human participants were reviewed and approved by the Local Ethical Review Board of Beijing Chaoyang Hospital and Sinopharm Tongmei General Hospital. The patients/participants provided their written informed consent to participate in this study.

## Author Contributions

LL and LX conceived the present study, participated in the design, conducted data analysis, and drafted the manuscript. XD and CL collected and assembled all data and commented on the manuscript drafts. LW and LX provided material and technical support and commented on the manuscript drafts. KL and XY aided in the interpretation of data, commented on the study design, and provided a critical review. All authors have read and approved the manuscript.

## Conflict of Interest

The authors declare that the research was conducted in the absence of any commercial or financial relationships that could be construed as a potential conflict of interest.

## Publisher's Note

All claims expressed in this article are solely those of the authors and do not necessarily represent those of their affiliated organizations, or those of the publisher, the editors and the reviewers. Any product that may be evaluated in this article, or claim that may be made by its manufacturer, is not guaranteed or endorsed by the publisher.
