# Supplementary material for: Feasibility and Safety of Drug-Coated Balloon-Only Angioplasty for De Novo Ostial Lesions of the Left Anterior Descending Artery: Two-Center Retrospective Study
Source: Front Cardiovasc Med. 2022 Apr 25;9:874394. doi: 10.3389/fcvm.2022.874394 (PMC9084228; doi:10.3389/fcvm.2022.874394)
Supplement: Supplementary file 1 [file Table_1.DOCX]

Supplementary table. the names of stents implanted in this study

| Factor | DES group |
| --- | --- |
| N | 49 |
| Drug-eluting stent type |  |
| Resolute Integrity | 20 (41%) |
| Xience Xpedition | 18 (37%) |
| Promus premier | 2 (4%) |
| Excel | 9 (18%) |

.
